# Physiological Study on Association between Nicotinamide N-Methyltransferase Gene Polymorphisms and Hyperlipidemia

**DOI:** 10.1155/2016/7521942

**Published:** 2016-11-23

**Authors:** Xiao-Juan Zhu, Ya-Jun Lin, Wei Chen, Ya-Hui Wang, Li-Qiang Qiu, Can-Xin Cai, Qun Xiong, Fei Chen, Li-Hui Chen, Qiong Zhou, Jiang-Hua Li

**Affiliations:** ^1^Key Lab of Training, Monitoring and Intervention of Aquatic Sports of General Administration of Sport of China, Institute of Physical Education, Jiangxi Normal University, Nanchang, China; ^2^Key Laboratory of Functional Small Organic Molecule, Ministry of Education, Jiangxi Normal University, Nanchang, China

## Abstract

Nicotinamide N-methyltransferase (NNMT) catalyzes the methylation of nicotinamide. Our previous works indicate that NNMT is involved in the body mass index and energy metabolism, and recently the association between a SNP (rs694539) of* NNMT* and a variety of cardiovascular diseases was reported. At present, more than 200* NNMT* single nucleotide polymorphisms (SNPs) have been identified in the databases of the human genome projects; however, the association between rs694539 variation and hyperlipidemia has not been reported yet, and whether there are any SNPs in* NNMT* significantly associated with hyperlipidemia is still unclear. In this paper, we selected 19 SNPs in* NNMT* as the tagSNPs using Haploview software (Haploview 4.2) first and then performed a case-control study to observe the association between these tagSNPs and hyperlipidemia and finally applied physiological approaches to explore the possible mechanisms through which the* NNMT* polymorphism induces hyperlipidemia. The results show that a SNP (rs1941404) in* NNMT* is significantly associated with hyperlipidemia, and the influence of rs1941404 variation on the resting energy expenditure may be the possible mechanism for rs1941404 variation to induce hyperlipidemia.

## 1. Introduction

Nicotinamide N-methyltransferase (NNMT) catalyzes the methylation of nicotinamide (NAM) using S-adenosylmethionine (SAM) as a methyl donor to generate methylnicotinamide (MNA) [1, 2]. The metabolic process and products of nicotinamide are highly related to various cardiovascular diseases, such as coronary heart disease, stroke, atherosclerosis, and diabetes [3, 4], which are closely related to an energy metabolism imbalance or obesity. Our previous studies showed that the nicotinamide metabolism rate was significantly correlated with the body mass index (BMI) [5] and energy metabolism [6], and these works have recently been supported by multiple reports. Kraus et al. [7] reported that NNMT expression is increased in white adipose tissue (WAT) and liver of obese and diabetic mice, and NNMT knockdown in WAT and liver protects against diet-induced obesity by increasing cellular energy expenditure. Liu et al. [8] found that serum MNA was associated with obesity and diabetes. Hong et al. [9] showed that nicotinamide N-methyltransferase regulated hepatic nutrient metabolism. Additionally, when NNMT catalyzes the methylation of nicotinamide, S-adenosyl homocysteine (SAH) and homocysteine (Hcy) are generated [10]. Hyperhomocysteinemia is one of the independent risk factors for cardiovascular diseases [11]; therefore, the relationship between NNMT and cardiovascular diseases has recently been increasingly reported [12–14].

Since the expression of NNMT is directly determined by* NNMT*, the roles of* NNMT* in the development of cardiovascular diseases have been reported in recent years. Bubenek et al. [14] reported that the occurrence and development of peripheral arterial occlusive diseases were closely related to* NNMT* expression and the serum NNMT level, and the* NNMT* expression level was significantly positively correlated with the low density lipoprotein level and significantly negatively correlated with the high density lipoprotein level. At present, more than 200* NNMT* single nucleotide polymorphisms (SNPs) in noncoding region have been identified in the human genome projects, but only one SNP (rs694539) has been reported in the genetic association studies. Souto et al. [11] reported that rs694539 variation is significantly associated with serum Hcy level in a Spanish population. Later, the associations between a variety of cardiovascular diseases and rs694539 variation were reported. van Driel et al. [15] reported that the risk of congenital heart diseases increased by eightfold in rs694539 AG+GG carriers under the conditions of low nicotinamide intake and drug exposure. Giusti et al. [16] reported that rs694539 variation was related to abdominal aortic diseases. de Jonge et al. [17] reported the association of rs694539 variation with lymphoblastic leukemia in children. Sazci et al. [18–20] reported the association of rs694539 variant with nonalcoholic steatohepatitis, bipolar disorder, and epilepsy, respectively. However, the association between rs694539 variation and hyperlipidemia has not been reported yet, and whether there are any SNPs in* NNMT* significantly associated with hyperlipidemia is still unclear.

Generally, hyperlipidemia is thought to be a major factor to induce many cardiovascular diseases. In this paper, we selected 19 SNPs (including rs694539) as the tagSNPs from* NNMT* DNA sequence in the database of 1000 Genomes Project using Haploview software (Haploview 4.2) first and then performed a case-control study to observe the association between these tagSNPs and hyperlipidemia and finally applied physiological approaches to explore the possible mechanisms through which* NNMT* polymorphism induces hyperlipidemia.

## 2. Subjects and Methods

### 2.1. Subjects

Cases and controls were all recruited from unrelated Chinese Han ethnicity volunteers. The investigation was approved by the local ethics committee at Jiangxi Normal University, and all participants gave the written informed consent. This study conforms to the latest revision of the Declaration of Helsinki.

The demographic and clinical characteristics of the 395 hyperlipidemic patients and the 316 controls are reported in Table 1. Usually, levels of plasma lipids increase with age, but after the age of 60 years, the trend is reversed. Levels of plasma lipids decrease with the increasing of age in the people aged ≥ 60 years. If a person aged ≥ 60 years has never had hyperlipidemia, then he/she has little chance to be of hyperlipidemia in the future, which means those people are the real nonsusceptible population to hyperlipidemia. Therefore, we recruited the controls from the volunteers aged ≥ 60 years. Meantime, simple hyperlipidemia usually occurs before the age of 60 years, and most hyperlipidemic patients aged ≥ 60 years have one or more related diseases, such as hypertension and diabetes. To enroll simple hyperlipidemic patients, we recruited the cases from the hyperlipidemic patients aged < 60 years. Finally, 395 patients aged < 60 years with simple hyperlipidemia (physician diagnosis, a recorded serum total cholesterol level > 220 mg/dL, and/or serum triglyceride level > 150 mg/dL) and 316 controls who were aged ≥ 60 years and had never been diagnosed as hyperlipidemia were enrolled. Exclusion criteria for the cases were poorly controlled diabetes mellitus (blood glucose > 6.67 mmol/L and/or glycosylated hemoglobin > 6.0%) and a possible secondary dyslipidemia, including thyroid and liver disease, renal failure, and proteinuria [21]; exclusion criteria for the controls were diabetes mellitus (hypoglycemic treatment and/or fasting blood glucose > 7 mmol/L), hypertension (hypertension treatment and/or blood pressure > 140/60 mmHg), or current treatment with lipid-affecting drugs [21].

To explore the roles of* NNMT* SNPs in the occurrence of hyperlipidemia, after the case-control studies, in which the SNPs significantly associated with hyperlipidemia and the susceptible genotypes were observed, we recruited an additional 150 healthy Chinese Han ethnicity male college students (aged 17–23 years and without any diagnosed diseases) to genotype the same SNPs. Then, these 150 subjects were divided into the susceptible group and the nonsusceptible group according to their genotypes and carried out the physiological studies.

### 2.2. Database Query and Determination of Investigation Sites

**Figure 1:**
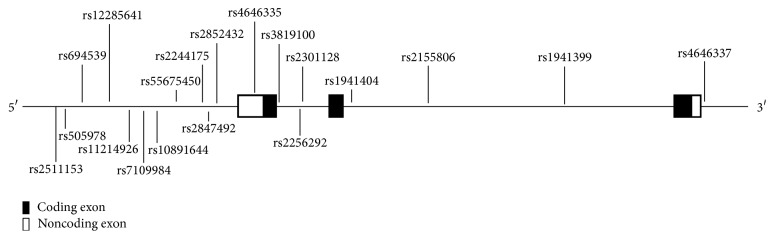
Positions of the 19 tagSNPs in relation to* NNMT*.

Nineteen* NNMT* tagSNPs were selected from the 208 known genotyped SNPs occurring within the Chinese Han population (CHB+CHS), contained within the database of the 1000 Genomes Project (http://browser.1000genomes.org/), with Haploview software (Haploview 4.2) after configuring the criteria (MAF > 0.10 and *r* ^2^ > 0.8) in this study. The positions of these 19 tagSNPs in relation to* NNMT* are shown in Figure 1.

### 2.3. Gene Polymorphism Detection

#### 2.3.1. Main Instruments and Reagents

The main instruments included a PCR machine (Norwalk, CT. 06859 USA), an electrophoresis apparatus (Beijing Junyi Electrophoresis Co., Ltd.), an automatic UV-Visible spectrometer, a biological electrophoresis image analysis system (Shanghai Furi Science and Technology Co., Ltd.), and a sequencer (ABI). The main reagents included PCR primers, dNTPs (Shanghai Hanyu Biological Engineering Co., Ltd.), a Taq enzyme system, ddH_2_O (Shanghai Biowing Applied Biotechnology Co., Ltd.), 1.5 mL Eppendorf tubes, pipette tips of all sizes, and 96-well PCR plates (Haimen Yonghui Experimental Equipment Co., Ltd.).

#### 2.3.2. DNA Extraction and Primer and Probe Design

Venous blood was collected, and genomic DNA was extracted using a DNA extraction kit (Promega, USA) and then stored at −80°C for future use. The NNMT gene sequence was downloaded from the NCBT database. Primers and probes were designed using Primer 3 online (Version 0.4.0, http://bioinfo.ut.ee/primer3-0.4.0/).

#### 2.3.3. SNP Genotyping

Polymerase chain reaction-ligase detection reaction (PCR-LDR) was used to detect the genotypes of each SNP. The principle of PCR-LDR is to use a high-temperature ligase to recognize gene polymorphism loci. PCR-LDR first utilizes a multiplex PCR to obtain gene fragments that contain the mutation sites to be measured, after which a multiplex LDR is performed, and finally, the fluorescent products of LDR were differentiated by ABI sequencer (PRISM 3730). To check the reliability of the genotyping results, 10% of PCR-LDR reactions were carried out in duplicate, and more than 99.5% had matching results. Additionally, 50 samples were randomly selected to genotype the most significantly associated SNP (rs1941404) identified in the association analysis using Sanger sequencing method, and 100% matched up perfectly with the results obtained with PCR-LDR method.

### 2.4. Physiological Tests

#### 2.4.1. Body Fat Percentage (BF%)

BF% was measured with bioimpedance measurement using an X-SCAN PLUS body composition analyzer (X-SCAN PLUS II, Jawon Medical Co., Ltd., Republic of Korea). Tests were carried out in the morning on an empty stomach.

#### 2.4.2. Resting Energy Expenditure (REE), Resting Energy Expenditure per Unit Body Surface Area (REEU), and Respiratory Quotient (RQ)

150 subjects were randomly selected from Chinese Han male college students to carry out a respiratory gas exchange analysis using a computerized indirect calorimetry with a ventilated-hood system (Metalyzer 3B, CORTEX Biophysik GmbH, Leipzig, Germany), as described previously [22], but one of them quitted for personal reason, and only 149 of them completed the test. The measurements were performed in a quiet room at 24°C, between 8:00 and 9:00 a.m., after an overnight fast of ≥12 h. The subjects sat in chairs quietly for 30 min before measurements were started and kept their position until measurements were completed. REE was calculated from measured oxygen consumption and carbon dioxide production according to the Weir equation: REE = 3.9 × O_2_ used + 1.1 × CO_2_ produced [23]. REEU was calculated as REE divided by body surface area (BSA), which was calculated using the Stevenson equation [24]: BSA (m^2^) = 0.0061 × height (cm) + 0.0128 × weight (kg) − 0.1529. RQ was calculated as VCO_2_/VO_2_.

### 2.5. Statistics

The distributions of allele and genotype frequencies, Hardy-Weinberg equilibrium (HWE), were conducted online using http://analysis.bio-x.cn/ [25]. Two classification logistic regressions were performed in the analyses of genotype effects and genetic models. The values of continuous variables were expressed as the mean value ± standard deviation (SD) and compared with independent *t*-test using IBM SPSS Statistics 20.0 (SPSS Inc., Chicago, IL, USA). All *P* values were two-sided, and values < 0.05 were considered to be statistically significant. Bonferroni correction was performed for multiple comparisons, and the corrected significance level was *P* < 0.0026.

## 3. Results and Discussion

### 3.1. NNMT Genotyping and Allele Frequency Analysis

The distributions of the genotype and allele frequencies of the 19 SNPs between the case and control groups are shown in Table 2. There are 5 SNPs (rs694539, rs12285641, rs11214926, rs2244175, and rs4646335) showing *P* < 0.05 in the HWE tests on the control group, which means those 5 SNPs are not in HWE and should not be considered in genetic association studies. Among the other 14 SNPs, which are in HWE (*P* > 0.05), 3 of them (rs10891644, rs4646335, and rs1941404) show *P* < 0.05 in the comparisons of genotype frequencies. However, after Bonferroni correction, only rs1941404 shows the corrected significant differences between the groups (*P* < 0.0026), which indicates that rs1941404 is the most significantly associated tagSNP in* NNMT* with hyperlipidemia. At this locus, the case group exhibits a higher frequency of allele C and a higher frequency of genotype CC than those of the control group, respectively.

Usually, when a locus is not in HWE, then this suggests deviations from random mating, population substructure, migration, natural selection, small population sizes, or genotyping errors. Considering that the genotyping results of this study have been verified with 10% samples tested in duplicate and validated using Sanger sequencing method, genotyping errors can be excluded, but what exactly makes the departure from HWE at those 5 SNPs is still unclear. Therefore, those 5 SNPs were not considered for further analysis. As mentioned previously, rs694539 is the most studied locus in* NNMT* and has been reported to be associated with many cardiovascular diseases. However, in this study, the distributions of genotypes and allele frequencies of rs694539 do not show significant differences (*P* < 0.0026) between the case and control groups (Table 2). The distribution of SNP allele frequencies has obvious population differences. Existing reports on the association between rs694539 variation and the diseases all employed a Spanish, Japanese, or Israeli population as the research subjects, whereas this study recruited a Chinese Han population. The differences in the study subjects might be the main reason for the different results obtained in the existing reports and this study. Moreover, although many diseases have been reported to be associated with rs694539, the association between rs694539 and hyperlipidemia has not been reported yet, suggesting that possibly the rs694539 is not the SNP which has the most significant regulatory effects on hyperlipidemia. Therefore, we do not analyze rs694539 further, and the subsequent analyses are primarily focused on rs1941404.

### 3.2. Genotype Effects of the rs1941404 Variation on Hyperlipidemia

Genotype effects usually are analyzed using the genetic models, recessive, dominant, additive, and codominant. However, these genetic models are not independent; sometimes several genetic models show the significance (*P* < 0.05) at the same time. To avoid the hash of possible genetic models and precisely determine the inheritance mode, Zintzaras and Santos [26] provided a whole solution and introduced the degree of dominance (*h*). As shown in Table 3, the chances of the subjects with the genotypes CC, CT, and TT to have hyperlipidemia are 70%, 48%, and 50%, which demonstrates that the homozygote CC has the greatest risk for hyperlipidemia and the risks of the genotypes TT and CT are almost equal, thus indicating that the genotype effects of the rs1941404 variation are a recessive inheritance mode. The results of two classification logistic regression analyses (Table 3) show that the recessive model and additive model are significant (*P* < 0.05), which further confirms that the genotype effects of the rs1941404 variation are a recessive inheritance mode, because the differences between the homozygote CC and TT carriers are significant (*P* < 0.05) and the OR values show that the risk to be hyperlipidemia for the CC is 2.432 and 2.316 times higher than those for the TT+CT and the TT, respectively. Table 3 also shows that the codominant model is significant (*P* < 0.05) which suggests that the heterozygote CT may have a significantly different risk of being hyperlipidemia from the homozygote CC+TT, and the values of *h* (−1 < *h* < 0) show that the risk of disease for CT is in the middle of the two homozygotes and close to TT. Therefore, genotype effects of rs1941404 variation basically belong to a recessive inheritance mode, and the CC carriers are the hyperlipidemia susceptible population and the CT or TT carriers are the nonsusceptible population, and therefore in the following exploration of the mechanisms, the differences between the CC and the CT+TT will be focused on.

### 3.3. Physiological Exploration of the Possible Mechanisms

**Figure 2:**
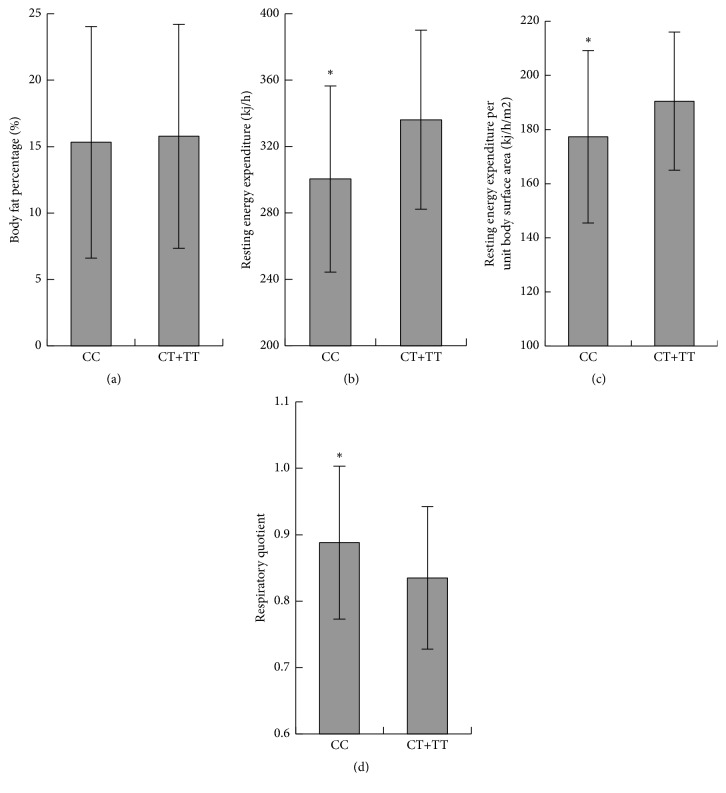
Effects of rs1941404 variation on the physiological indicators. (a) Body fat percentage; (b) resting energy expenditure rate; (c) unit surface area resting energy expenditure rate; (d) respiratory quotient. CC, the genotype CC (*n* = 28); CT+TT, the genotypes (CT+TT) (*n* = 121). Error bars, ± standard deviation. ^*∗*^ *P* < 0.05 compared to CT+TT.

Medically, hyperlipidemia is the one symptom of metabolic syndrome, which is thought to be caused by an underlying disorder of energy utilization and storage [27], so that BF% and the characters of the resting energy expenditure (REE) may be able to offer some clues for the exploration of the possible mechanisms, through which rs1941404 variation induces the hyperlipidemia. As shown in Figure 2, BF% of the CC carriers is substantially equivalent to that of the (CT+TT) carriers (*P* > 0.05). However, the REE and REEU of the CC genotype are significantly lower than those of the (CT+TT) genotype (*P* < 0.05) and the RQ is significantly higher than that of the (CT+TT) genotypes (*P* < 0.05).

Theoretically, it is not surprising that* NNMT* variation has a great impact on an organism's energy metabolism. The physiological function of NNMT is to transfer a methyl group from SAM and catalyze the methylation of NAM. Firstly, NAM is the precursor of NAD^+^. NAM methylation directly affects the level of NAD^+^, which is the key coenzyme for energy metabolism and is directly involved in the entire process of aerobic oxidation. Therefore, changes in NAD^+^ level will directly affect the energy metabolism process. Secondly, the changes of NNMT level may indirectly affect the expression of various related proteins. For example, Zhang et al. analyzed the SW480 cell lines before and after NNMT transfection [28] and found that more than 30 genes demonstrated greater than twofold differences in gene expression before and after transfection, of which most genes were related to glucose and lipid metabolism and the oxidative respiratory chain.

Epidemiological studies have shown that the hyperlipidemia has a significant association with obesity or energy metabolism imbalance. The results of the present study suggest that although the BF% of the CC genotype subjects was not higher than that of the CT+TT genotype subjects (Figure 2(a)), the probability of the CC genotype subjects to be fat in the future is higher than that of the CT+TT genotype subjects, because the CC genotype subjects have the lower REE and REEU values (Figures 2(b) and 2(c)), which are the most important factors leading to obesity. The RQ value reflects the proportion of the energy supply from different energy materials. The RQ value of the CC genotype subjects is higher and very close to 1, which indicates that before the appearance of the hyperlipidemia these subjects utilize a relatively high proportion of glucose to generate energy other than lipids. Therefore, the effects of rs1941404 variations on REE may be a primary physiological mechanism leading to the development of the hyperlipidemia.

## 4. Conclusion

The rs1941404 variation in* NNMT* is significantly associated with hyperlipidemia and the CC is the risk genotype (recessive inherence mode). The influences of rs1941404 variation on the resting energy expenditure may be the possible physiological mechanisms for rs1941404 variation to induce hyperlipidemia.

## Figures and Tables

**Table 1:** Demographic and clinical characteristics of hyperlipidemic patients and controls.

	Controls (*n* = 316)	Patients (*n* = 395)	Significance
Age (years)	71.48 ± 7.90	49.59 ± 8.81	*P* < 0.001
Gender (male/female)	175/141	194/201	*P* = 0.113
BMI (kg/m^2^)	22.63 ± 4.51	24.98 ± 4.23	*P* < 0.01
Triglycerides (mg/dL)	110.88 ± 57.64	267.66 ± 129.71	*P* < 0.001
Total cholesterol (mg/dL)	196.97 ± 35.89	251.97 ± 57.15	*P* < 0.001
LDL cholesterol (mg/dL)	119.06 ± 30.42	161.528 ± 51.22	*P* < 0.001
HDL cholesterol (mg/dL)	57.14 ± 12.87	47.72 ± 15.41	*P* < 0.001
Blood glucose (mmol/L)	5.14 ± 1.18	5.37 ± 1.25	*P* < 0.05
Diabetes, *n* (%)	0 (0%)	76 (19.24%)	*P* < 0.001
Hypertension, *n* (%)	0 (0%)	55 (13.92%)	*P* < 0.001

BMI, body mass index; LDL, low density lipoprotein; HDL, high density lipoprotein.

**Table 2:** Frequency distributions of the hyperlipidemia cases and controls.

SNPs	Allele			*P*	Genotype			HWE	*P*
rs2511153	Case	C: 525 (0.68)	T: 253 (0.33)	0.035	CC: 172 (0.44)	CT: 181 (0.47)	TT: 36 (0.09)		0.095
	Ctrl	C: 390 (0.62)	T: 238 (0.38)		CC: 116 (0.37)	CT: 158 (0.50)	TT: 40 (0.13)	0.22	

rs505978	Case	A: 443 (0.59)	G: 309 (0.41)	0.067	AA: 131 (0.35)	AC: 181 (0.48)	CC: 64 (0.17)		0.115
	Ctrl	A: 338 (0.54)	G: 288 (0.46)		AA: 86 (0.28)	AC: 166 (0.53)	CC: 61 (0.20)	0.23	

rs694539	Case	A: 262 (0.35)	G: 494 (0.65)	0.665	AA: 54 (0.14)	AG: 154 (0.41)	GG: 170 (0.45)		0.005
	Ctrl	A: 210 (0.34)	G: 416 (0.67)		AA: 25 (0.08)	AG: 160 (0.51)	GG: 128 (0.41)	0.01	

rs12285641	Case	C: 457 (0.58)	T: 325 (0.42)	0.090	CC: 139 (0.36)	CT: 179 (0.46)	TT: 73 (0.19)		0.010
	Ctrl	C: 390 (0.63)	T: 230 (0.37)		CC: 113 (0.37)	CT: 164 (0.53)	TT: 33 (0.11)	0.02	

rs11214926	Case	A: 242 (0.32)	G: 514 (0.68)	0.013	AA: 48 (0.13)	AG: 146 (0.39)	GG: 184 (0.49)		0.001
	Ctrl	A: 162 (0.26)	G: 464 (0.74)		AA: 12 (0.04)	AG: 138 (0.44)	GG: 163 (0.52)	0.01	

rs7109984	Case	C: 680 (0.87)	T: 102 (0.13)	0.652	CC: 290 (0.74)	CT: 100 (0.26)	TT: 1 (0.00)		0.271
	Ctrl	C: 534 (0.86)	T: 86 (0.14)		CC: 228 (0.74)	CT: 78 (0.25)	TT: 4 (0.01)	0.35	

rs10891644	Case	G: 509 (0.65)	T: 269 (0.35)	0.109	GG: 158 (0.41)	GT: 193 (0.50)	TT: 38 (0.10)		0.011
	Ctrl	G: 428 (0.70)	T: 188 (0.31)		GG: 155 (0.50)	GT: 118 (0.38)	TT: 35 (0.11)	0.09	

rs55675450	Case	A: 119 (0.15)	G: 659 (0.85)	0.976	AA: 13 (0.03)	AG: 93 (0.24)	GG: 283 (0.73)		0.987
	Ctrl	A: 96 (0.15)	G: 534 (0.85)		AA: 11 (0.04)	AG: 74 (0.24)	GG: 230 (0.73)	0.11	

rs2244175	Case	A: 404 (0.53)	G: 352 (0.47)	0.117	AA: 103 (0.27)	AG: 198 (0.52)	GG: 77 (0.20)		0.115
	Ctrl	A: 308 (0.49)	G: 318 (0.51)		AA: 64 (0.20)	AG: 180 (0.58)	GG: 69 (0.22)	0.01	

rs2847492	Case	A: 264 (0.35)	G: 492 (0.65)	0.882	AA: 51 (0.14)	AG: 162 (0.43)	GG: 165 (0.44)		0.179
	Ctrl	A: 221 (0.35)	G: 405 (0.65)		AA: 33 (0.11)	AG: 155 (0.50)	GG: 125 (0.40)	0.14	

rs2852432	Case	C: 399 (0.53)	T: 357 (0.47)	0.022	CC: 101 (0.27)	CT: 197 (0.52)	TT: 80 (0.21)		0.051
	Ctrl	C: 369 (0.59)	T: 257 (0.41)		CC: 102 (0.33)	CT: 165 (0.53)	TT: 46 (0.15)	0.11	

rs4646335	Case	A: 487 (0.64)	T: 269 (0.36)	0.011	AA: 161 (0.43)	AT: 165 (0.44)	TT: 52 (0.14)		0.004
	Ctrl	A: 360 (0.58)	T: 264 (0.42)		AA: 95 (0.30)	AT: 170 (0.55)	TT: 47 (0.15)	0.04	

rs3819100	Case	A: 431 (0.57)	G: 325 (0.43)	0.005	AA: 120 (0.32)	AG: 191 (0.51)	GG: 67 (0.18)		0.012
	Ctrl	A: 309 (0.49)	G: 317 (0.51)		AA: 70 (0.22)	AG: 169 (0.54)	GG: 74 (0.24)	0.16	

rs2256292	Case	C: 327 (0.43)	G: 439 (0.57)	0.053	CC: 71 (0.19)	CG: 185 (0.48)	GG: 127 (0.33)		0.063
	Ctrl	C: 236 (0.38)	G: 392 (0.62)		CC: 38 (0.12)	CG: 160 (0.51)	GG: 116 (0.37)	0.13	

rs2301128	Case	A: 100 (0.13)	G: 678 (0.87)	0.739	AA: 9 (0.02)	AG: 82 (0.21)	GG: 298 (0.77)		0.924
	Ctrl	A: 77 (0.12)	G: 551 (0.88)		AA: 6 (0.02)	AG: 65 (0.21)	GG: 243 (0.77)	0.50	

*rs1941404*	Case	C: 391 (0.53)	T: 349 (0.47)	*0.000*	CC: 117 (0.32)	CT: 157 (0.42)	TT: 96 (0.26)		*0.000*
	Ctrl	C: 268 (0.43)	T: 358 (0.57)		CC: 50 (0.16)	CT: 168 (0.54)	TT: 95 (0.30)	*0.09*	

rs2155806	Case	C: 78 (0.10)	T: 700 (0.90)	0.346	CC: 7 (0.01)	CT: 64 (0.17)	TT: 318 (0.82)		0.322
	Ctrl	C: 73 (0.12)	T: 557 (0.88)		CC: 4 (0.01)	CT: 65 (0.21)	TT: 246 (0.78)	0.90	

rs1941399	Case	A: 119 (0.16)	C: 637 (0.84)	0.550	AA: 7 (0.02)	AC: 105 (0.28)	CC: 266 (0.70)		0.525
	Ctrl	A: 106 (0.17)	C: 520 (0.83)		AA: 10 (0.03)	AC: 86 (0.28)	CC: 217 (0.69)	0.68	

rs4646337	Case	A: 680 (0.88)	G: 94 (0.12)	0.748	AA: 296 (0.77)	AG: 88 (0.23)	GG: 3 (0.01)		0.250
	Ctrl	A: 557 (0.88)	G: 73 (0.12)		AA: 248 (0.79)	AG: 61 (0.19)	GG: 6 (0.02)	0.33	

Case, the hyperlipidemia group; Ctrl, the control group; HWE, *P* value of Hardy-Weinberg equilibrium test on the control group; the values of allele and genotype are the number of individuals (frequency).

**Table 3:** Genotype effects and genetic models of rs1941404 variation.

Model	Genotype	Case	Control	OR (95% CI)	*P*	*h*
Recessive	CC	117 (0.70)	50 (0.30)	2.432 (1.574, 3.534)	*0.000*	−0.54
	TT+CT	253 (0.49)	263 (0.51)			
Dominant	CC+CT	274 (0.56)	218 (0.44)	1.244 (0.890, 1.738)	0.202	
	TT	96 (0.50)	95 (0.50)			
Additive	CC	117 (0.70)	50 (0.38)	2.316 (1.497, 3.581)	*0.000*	
	TT	96 (0.50)	95 (0.59)			
Codominant	CT	157 (0.48)	168 (0.52)	0.636 (0.470, 0.861)	*0.003*	
	CC+TT	213 (0.59)	145 (0.41)			

Case, the hyperlipidemia group; Ctrl, the control group; the values of the case and control are the number of individuals (frequency) from different genotypes; OR, odds ratio; CI, confidence interval; *h* (dominance degree) = ln⁡(OR_co_)/ln⁡(OR_*a*_), OR_co_, OR of the codominant model; OR_*a*_, OR of the additive model.
